# Developing a CT radiomics-based model for assessing split renal function using machine learning

**DOI:** 10.1007/s11604-025-01786-6

**Published:** 2025-06-03

**Authors:** Yihua Zhan, Junjiong Zheng, Xutao Chen, Yushu Chen, Chao Fang, Cong Lai, Mingzhou Dai, Zhikai Wu, Han Wu, Taihui Yu, Jian Huang, Hao Yu

**Affiliations:** 1 The Sun Yat-Sen Memorial Hospital of Sun Yat-Sen University, Guangzhou, China; 2 The Eighth Affiliated Hospital of Sun Yat-Sen University, Shenzhen, China; 3 The Education University of Hong Kong, Hong Kong, China

**Keywords:** Split renal function, Radiomics, Machine learning, Assessment model, Non-contrast computed tomography

## Abstract

**Purpose:**

This study aims to investigate whether non-contrast computed tomography radiomics can effectively reflect split renal function and to develop a radiomics model for its assessment.

**Materials and methods:**

This retrospective study included kidneys from the study center and split them into training (70%) and testing (30%) sets. Renal dynamic imaging was used as the reference standard for measuring split renal function. Based on chronic kidney disease staging, kidneys were categorized into three groups according to glomerular filtration rate: > 45 ml/min/1.73 m^2^, 30–45 ml/min/1.73 m^2^, and < 30 ml/min/1.73 m^2^.Features were selected based on feature importance ranking from a tree model, and a random forest radiomics model was built.

**Results:**

A total of 543 kidneys were included, with 381 in the training set and 162 in the testing set. In the training set, 16 features identified as most important for distinguishing between the groups were ultimately included to develop the random forest model. The model demonstrated good discriminatory ability in the testing set. The AUC for the > 45 ml/min/1.73 m^2^, 30–45 ml/min/1.73 m^2^, and < 30 ml/min/1.73 m^2^ categories were 0.859 (95% CI 0.804–0.910), 0.679 (95% CI 0.589–0.760), and 0.901 (95% CI 0.848–0.946), respectively. The calibration curves for the kidneys in each group closely align with the diagonal, with Hosmer–Lemeshow test *P*-values of 0.124, 0.241, and 0.199 for the three groups, respectively (all *P* > 0.05). The decision curve analysis confirmed the radiomics model's clinical utility, demonstrating significantly higher net benefit than both treat-all and treat-none strategies at clinically relevant probability thresholds: 1–69% and 71–75% for the > 45 ml/min/1.73 m^2^ group, 15-d50% for the 30–45 ml/min/1.73 m^2^ group, and 0–99% for the < 30 ml/min/1.73 m^2^ group.

**Conclusion:**

Non-contrast computed tomography radiomics can effectively reflect split renal function information, and the model developed based on it can accurately assess split renal function, holding great potential for clinical application.

## Introduction

The evaluation of Split Renal Function (SRF) entails the individual assessment of each kidney’s functionality. Precision in determining SRF is imperative for guiding clinical decisions.

Renal dynamic imaging (RDI) [1] is the predominant technique for the quantitative analysis of SRF in clinical practice, with measurements derived via the Gates [2] method deemed highly precise and widely accepted as the gold standard in numerous medical facilities. Nonetheless, RDI is encumbered by drawbacks such as low spatial resolution, prohibitive costs, time-consuming, and stringent equipment requirements [3, 4], which hinder its broad implementation, particularly in primary healthcare contexts.

Non-contrast computed tomography (NCT) provides a rapid and straightforward approach, with no contrast agent-related risks compared to contrast-enhanced CT and greater time efficiency and accessibility compared to magnetic resonance imaging, making it particularly suitable for urgent clinical situations. In addition, it features high spatial resolution, ease of operation, and cost-effectiveness, with minimal requirements for medical infrastructure and personnel, thereby offering broad applicability. Numerous studies have demonstrated that morphological information from renal NCT, such as renal volume, is correlated with SRF [5, 6]. However, the strength of these correlations remains controversial [7].

Radiomics [8], a nascent discipline merging medical imaging with comprehensive data analysis, extracts a multitude of quantitative attributes from imaging datasets. Through computational algorithmic analysis, radiomics can reveal subtleties in the data that may not be apparent to the naked eye, thus providing enhanced clinical insights [9]. Previous studies have highlighted the potential of radiomics in assessing SRF. Notably, a recent study demonstrated that magnetic resonance radiomics could predict SRF. Additionally, existing studies have validated the ability of NCT radiomics to assess overall renal function in patients [10, 11]. However, research on NCT radiomics for the prediction of SRF remains in the exploratory phase.

Therefore, the primary aim of this study is to explore the potential of NCT radiomics in reflecting SRF information and to develop a radiomics-based model for SRF assessment. This model is intended to provide a more streamlined and cost-effective approach for evaluating SRF, thereby offering a novel methodology for clinical application.

## Methods

### Clinical data collection

This study, conducted in a retrospective design, received ethical clearance from the Ethics Committee of the research center, where the requirement for informed consent was waived.

**Fig. 1:**
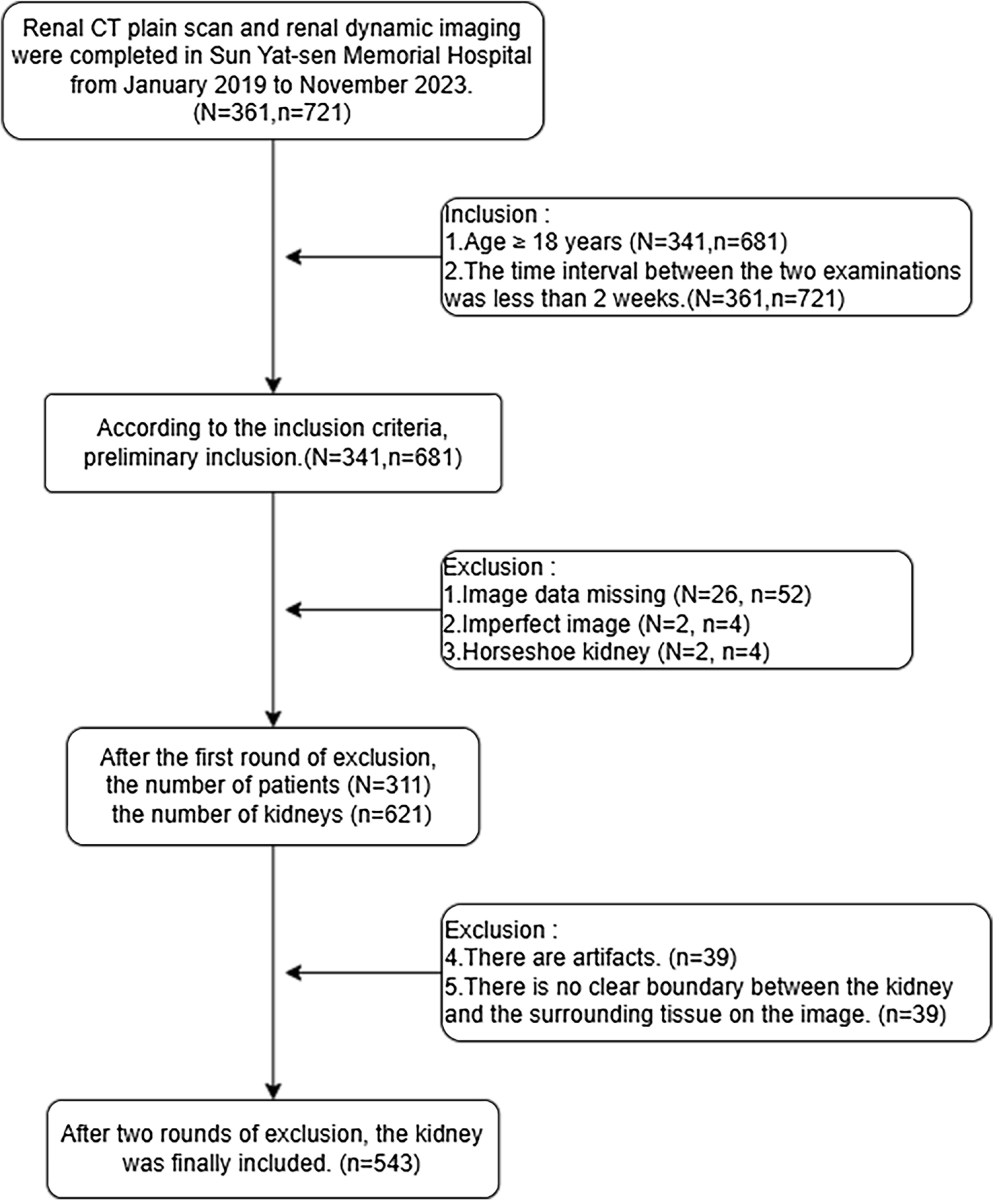
Patients and kidneys screening process. N: number of patients, *n*: number of kidneys (One man isolated kidney)

Clinical information and original computed tomography images were collated from a cohort of 341 patients who had undergone both renal NCT and RDI at Sun Yat-sen Memorial Hospital between January 2019 and November 2023. The dataset encompassed 543 kidneys, which were subsequently allocated to training and testing sets in a ratio of 7:3. The training set consisted of 381 kidneys, and the testing set comprised 162 kidneys. The patients and kidneys screening processes are shown in Fig. 1.

Inclusion criteria were as follows: (1) Renal NCT and RDI were performed concurrently between January 2019 and November 2023; (2) The time interval between the two examinations was less than 2 weeks; (3) Age ≥ 18 years. Exclusion criteria included: (1) Missing imaging data; (2) Incomplete images; (3) Horseshoe kidney; (4) Presence of artifacts in images; (5) Poor image quality, such as indistinct boundaries between the kidney and surrounding tissues.

This study used the SRF measured by RDI as the gold standard. Based on chronic kidney disease staging [12], kidneys were categorized into three groups according to glomerular filtration rate (GFR): > 45 ml/min/1.73 m^2^, 30–45 ml/min/1.73 m^2^, and < 30 ml/min/1.73 m^2^.

We further classified the kidneys based on CT imaging diagnosis into three groups—normal kidneys, obstructed kidneys, and space-occupying kidneys—for subgroup analysis. Normal kidneys were defined as those with no significant abnormalities on imaging (such as kidney stones, hydronephrosis, renal cysts, or renal tumors). Obstructed kidneys refer to those with upper urinary tract obstruction caused by various lesions, such as stones or tumors. Space-occupying kidney diseases included kidneys with cysts or tumors that did not cause urinary tract obstruction.

### Renal dynamic imaging data collection

RDI is a nuclear medicine examination, and its measurement of SRF was used as the gold standard in this study. The data collection method involved positioning the probe posteriorly, encompassing both the kidneys and the bladder region. A "bolus" injection of ^99^mTc-DTPA was administered via the antecubital vein at a dose of approximately 5 mCi, and data acquisition commenced when the tracer reached the abdominal aorta. The imaging was performed at a rate of 1 frame every 1–5 s for 60 s to capture renal perfusion information. Following this, continuous imaging was conducted at 1 frame per minute for 30 min to visualize tracer accumulation and excretion in the kidneys. By analyzing the uptake, processing, and excretion of the tracer by each kidney, the percentage of each kidney's function relative to total renal function was calculated, ultimately determining the SRF for each side.

### CT image acquisition and image segmentation

Imaging of all kidneys was conducted utilizing a Siemens third-generation 192-slice dual-source CT scanner (SOMATOM Force, Siemens Healthineers, Forchheim, Germany) or a GE 64-slice single-source CT scanner (Discovery CT750 HD, GE Healthcare, Pewaukee, WI, USA) available at the imaging center. The scanning parameters were configured as follows: an effective tube current of 350 mA, a tube voltage of 120 kV, a matrix size of 512*512, a field of view of 378 mm, a slice thickness of either 1.0 or 1.25 mm, and a slice spacing of 1.0 mm. The Siemens scanner utilized the ADMIRE iterative reconstruction technique with an iterative reconstruction strength level of 3 and a convolution kernel type of Br40. The GE scanner utilized the ASIR iterative reconstruction technique at a 50% strength level. The imaging was conducted with the patient in a supine position. Images were exported in DICOM format and then imported into 3D Slicer software [13] version 5.4.0 for image segmentation and feature extraction. To minimize image differences arising from different CT devices, all CT images were first resampled to a uniform voxel size of 1 mm * 1 mm * 1 mm using cubic spline interpolation for pixel size standardization.

This study primarily focuses on the renal parenchyma as the region of interest (ROI). During segmentation, the entire renal parenchyma should be included as comprehensively as possible, while excluding the renal pelvis, calyces, ureteral collecting system, blood vessels and fat within the renal sinus, perirenal fat, and any space-occupying lesions such as cysts and tumors within the renal parenchyma.

**Fig. 2:**
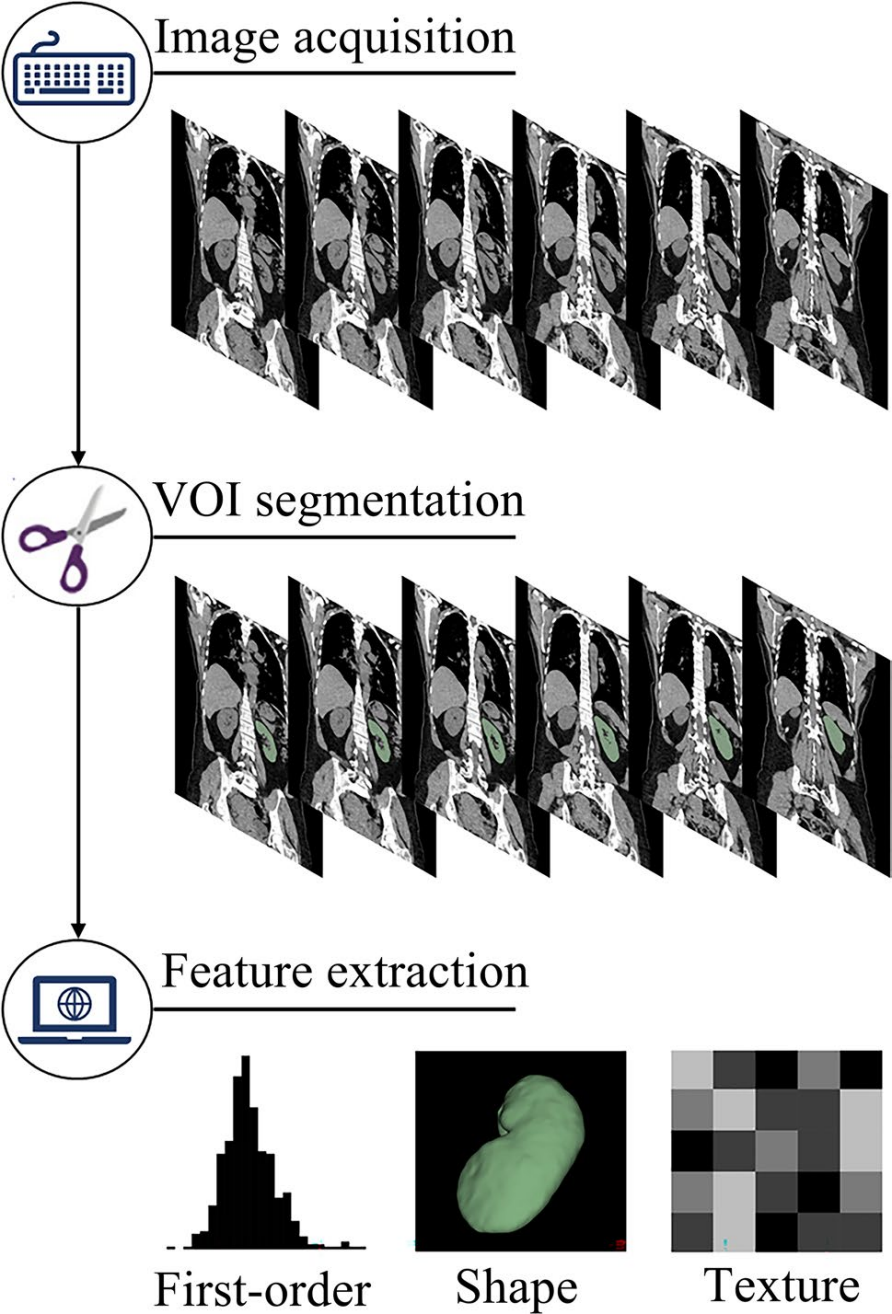
The workflow of radiomics. The radiomics workflow presents the procedure of radiomics analysis, consisting of 3 steps: image acquisition, volume of interest (VOI) segmentation, and feature extraction

The segmentation was performed by two experienced radiologists using the semi-automatic "Grow from Seeds" tool to delineate the ROI. The process involved placing green seeds on the axial, sagittal, and coronal planes to mark the renal parenchyma, ensuring at least three slices per plane were annotated for accuracy. Yellow seeds were also used to mark the renal parenchyma contours in the non-ROI areas, restricting seed growth. After seed placement, the "Initialize" function allowed seeds to grow in areas with similar pixel values, followed by manual adjustments for precision. Once the segmentation was satisfactory, the ROI was retained, and a 3D reconstruction of the ROI was generated. Finally, fine adjustments were made using tools like "Erase" and "Scissors" for further precision. The image segmentation and feature extraction process is shown in Fig. 2.

### Radiomics features extraction and features selection

Radiomic features from the renal parenchyma were extracted using the PyRadiomics tool [14] in 3D Slicer software, encompassing a total of 1316 features, including first-order features, texture features, and shape features. First-order features describe the fundamental characteristics of pixel intensity distributions, reflecting the overall brightness and contrast of the image, and serve as the foundational elements in radiomics analysis. Texture features, which capture the complexity and repetitiveness of the internal structures and patterns of the image, include the Gray-Level Co-occurrence Matrix (GLCM), Gray-Level Dependence Matrix (GLDM), Gray-Level Run-Length Matrix (GLRLM), Gray-Level Size Zone Matrix (GLSZM), and Neighborhood Gray-Tone Difference Matrix (NGTDM). Shape features describe the geometric shape and size of objects within the image. Additionally, features were subjected to Log filtering and three-dimensional Wavelet transformation to detect and enhance image edges and details, thereby extracting multi-scale texture features.

Initially, the extracted radiomic features were normalized using z-score transformation, converting them to a distribution with a mean of 0 and a standard deviation of 1. Spearman Correlation Coefficient was used to assess feature correlations, and features with correlation coefficients greater than 0.9 were reduced to one per group.

**Fig. 3:**
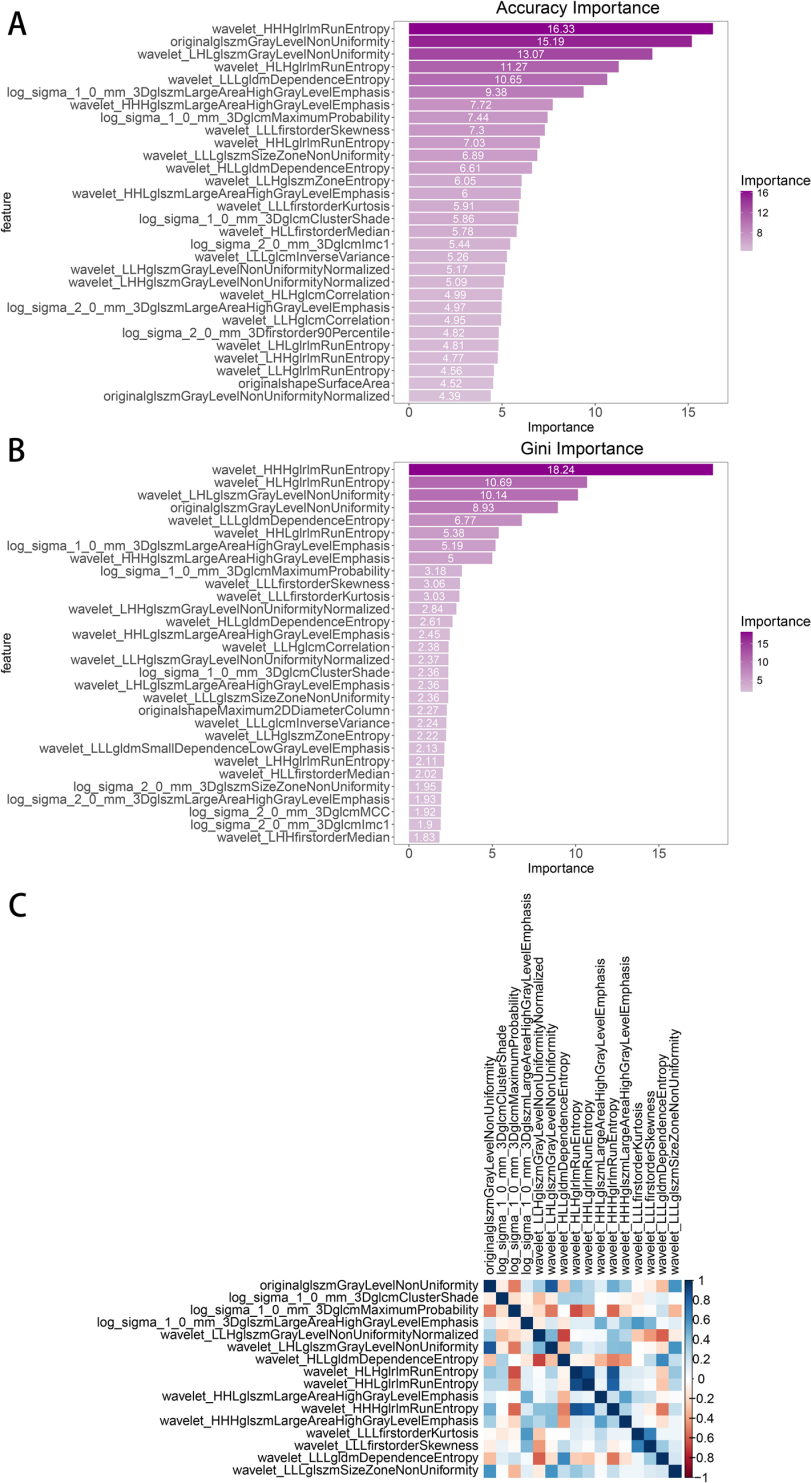
Features selection. **a**,**b** The histogram shows the importance ranking and its coefficients based on tree model features. Each feature is presented in the format of ' filter-feature class-feature name '. For the filter ' LoG ', the following numerical values represent the filter width for the Gaussian kernel. For the filter ' wavelet ', the following three uppercase letters represent the filters applied in the x, y, and z directions, respectively (H, high-pass filter; l, low-pass filter); shape, representing morphological features; first order, representing the first-order basic feature; glcm, represents the gray level co-occurrence matrix; gldm, represents the gray dependence matrix; glrlm, represents the gray run-length matrix; glszm, represents the gray-scale region matrix. **a** Accuracy feature importance is based on the contribution of features to the overall accuracy of the model. **b** Gini feature importance is based on the contribution of features to node Gini impurity. **c** The Spearman correlation heatmap of the 16 selected radiomics features is shown, where darker colors indicate stronger correlations. The color scale and corresponding correlation coefficients are displayed on the right. The results indicate that the correlation coefficients of these selected radiomics features are all less than 0.9

Finally, feature selection was performed based on feature importance within a tree-based model, retaining the intersection of the top 20 features ranked by Accuracy importance and Gini importance (Fig. 3a–b), ultimately selecting 16 radiomic features for model construction. Figure 3c shows the correlation heatmap of the 16 features used to construct the model, respectively.

### Radiomics model development and validation

The final selected radiomic features were used to construct a multi-class random forest model. The model was trained using tenfold cross-validation within the training set, with parameters mTry and nTree optimized to achieve the best performance. The model's generalization ability was then assessed in the testing set. Model performance was evaluated using Receiver Operating Characteristic (ROC) curves and the Area Under the Curve (AUC), as well as metrics such as accuracy, sensitivity, specificity, and F1 score. The reliability of the model was evaluated using the Calibration Curve, while the clinical Decision Curve Analysis was used to assess the practical utility of the model. Finally, the model’s generalization ability was evaluated in the testing set.

### Statistical analysis

All statistical analyses were conducted using R software (version 4.4.0) and Python (version 3.12.5). Quantitative data are presented as means (standard deviation) and medians [range], while qualitative data are expressed as frequencies (percentages). Comparisons of categorical variables were performed using the Chi-square test. Continuous variables were assessed using the t-test or analysis of variance. *P*-value < 0.05 was considered statistically significant. For the missing clinical baseline data, values were imputed using the mean.

## Results

### Clinical and demographic data

The basic clinical and demographic characteristics of the kidneys are summarized in Table 1. A total of 543 kidneys were included in the study, with 381 kidneys in the training set and 162 kidneys in the testing set. The groups with GFR > 45 ml/min/1.73 m^2^, 30–45 ml/min/1.73 m^2^, and < 30 ml/min/1.73 m^2^ included 178, 175, and 190 kidneys, respectively. Additionally, the subgroups of normal, obstructive, and space-occupying kidneys consisted of 153, 257, and 133 kidneys, respectively.

**Table 1:** Baseline characteristics of training set and testing set

	Training set	Testing set	ALL	P-value
	(*n* = 381)	(*n* = 162)	(*n* = 543)	
*Gender*				
male	195 (51.2%)	81 (50.0%)	276 (50.8%)	0.963
female	186 (48.8%)	81 (50.0%)	267 (49.2%)	
*Age*				
Mean (SD)	56.9 (13.1)	55.3 (13.4)	56.4 (13.2)	0.438
Median [Min, Max]	58.0 [20.0, 83.0]	57.0 [20.0, 84.0]	57.0 [20.0, 84.0]	
*Height*				
Mean (SD)	162 (7.10)	162 (6.87)	162 (7.03)	0.986
Median [Min, Max]	162 [145, 183]	162 [145, 187]	162 [145, 187]	
*Weight*				
Mean (SD)	61.3 (11.6)	60.4 (10.6)	61.1 (11.3)	0.632
Median [Min, Max]	61.0 [30.0, 105]	60.0 [36.0, 102]	61.0 [30.0, 105]	
*BMI*				
Mean (SD)	23.3 (3.71)	23.0 (3.17)	23.2 (3.56)	0.591
Median [Min, Max]	23.3 [13.3, 38.6]	22.9 [14.4, 30.8]	23.1 [13.3, 38.6]	
*Hypertension*				
yes	141 (37.0%)	51 (31.5%)	192 (35.4%)	0.487
no	240 (63.0%)	111 (68.5%)	351 (64.6%)	
*Diabetes*				
yes	62 (16.3%)	17 (10.5%)	79 (14.5%)	0.219
no	319 (83.7%)	145 (89.5%)	464 (85.5%)	
*GFR*				
> 45 ml/min/1.73m^2^	125 (32.8%)	53 (32.7%)	178 (32.8%)	1
30–45 ml/min/1.73m^2^	123 (32.3%)	52 (32.1%)	175 (32.2%)	
< 30 ml/min/1.73m^2^	133 (34.9%)	57 (35.2%)	190 (35.0%)	
*CT diagnosis*				
Normal kidneys	103 (27.0%)	50 (30.9%)	153 (28.2%)	0.926
Obstructive kidneys	184 (48.3%)	73 (45.1%)	257 (47.3%)	
Space-occupying kidneys	94 (24.7%)	39 (24.1%)	133 (24.5%)	
*Right/Left side*				
Right kidney	187 (49.1%)	88 (54.3%)	275 (50.6%)	0.535
Left kidney	194 (50.9%)	74 (45.7%)	268 (49.4%)	

Data are presented as *n* (%) unless indicated otherwise

P < 0.05 indicated that the difference was statistically significant

### The model evaluation performance

**Fig. 4:**
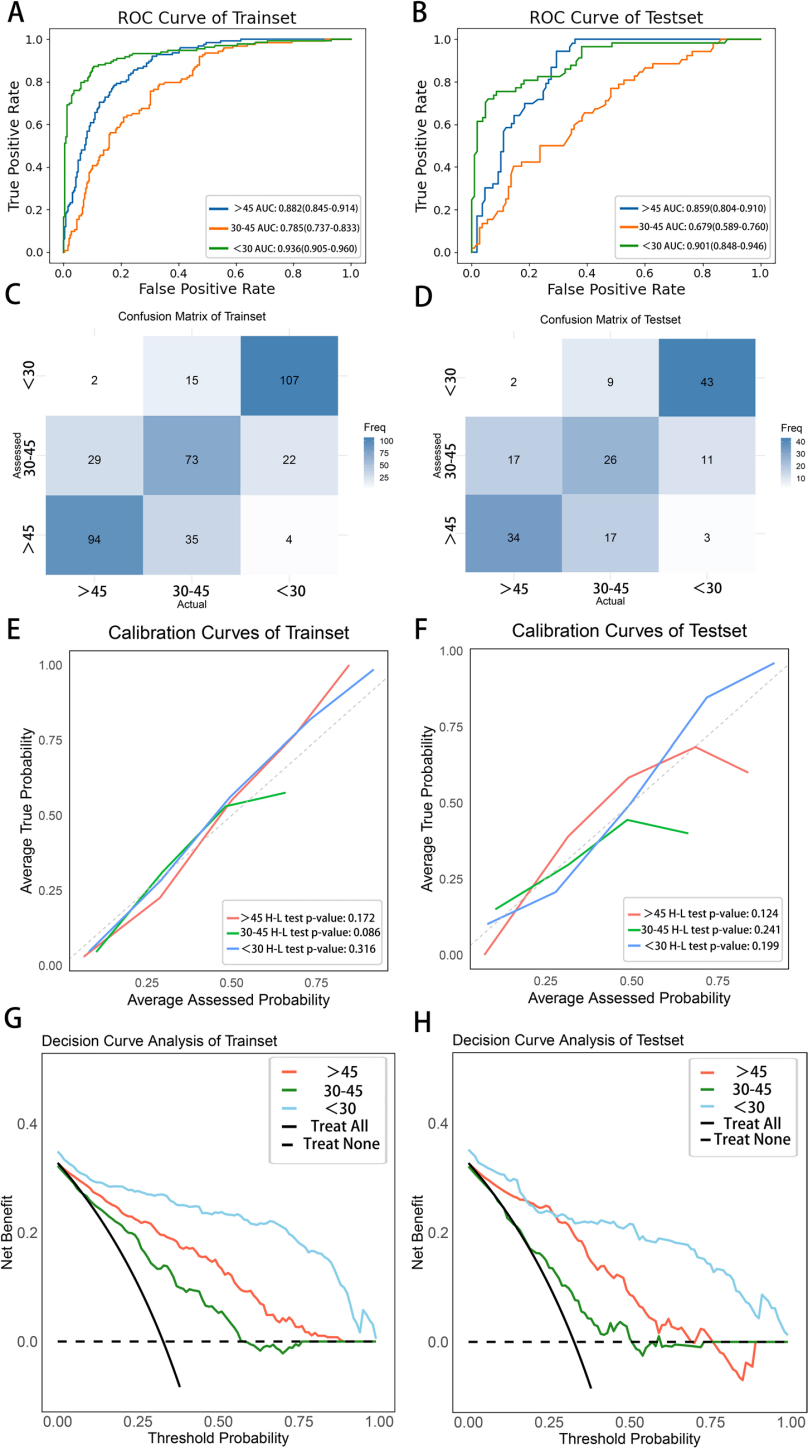
Performance of the radiomics model. **a**–**b** The receiver operating characteristic (ROC) curves of the three groups of the training set and the testing set of the radiomics prediction model were shown in Fig. 4**a**–**b**. > 45, 30 ~ 45, and < 30 represent the three groups of split renal function. **c–d** The confusion matrices of the training set and testing set are shown. The x-axis and y-axis showed the actual and assessed groups of split renal function, respectively. **e**–**f** Calibration curves of the radiomics model for the training and testing sets. The x-axis and y-axis represent the assessed and actual probabilities of split renal function, respectively. The three distinct curves, in different colors, correspond to kidneys with varying levels of function. The Hosmer–Lemeshow test, with *n*-bins set to 10, yielded a *P*-value > 0.05, indicating that the difference between the predicted and actual probabilities is not statistically significant, suggesting good agreement between the model’s predictions and the actual outcomes. **g–h** Decision curve analysis of the radiomics model for both the training and testing sets. Curves in different colors represent kidneys with different levels of function, while the black solid line assumes all kidneys receive treatment, and the black dashed line assumes no treatment is provided. The area between these colored curves and the two black lines reflects the clinical net benefit of implementing treatment based on the model’s assessed results, compared to the "treat all" or "treat none" strategies

The random forest model demonstrated overall good predictive ability. In the training set, the AUC for the > 45 ml/min/1.73 m^2^, 30–45 ml/min/1.73 m^2^, and < 30 ml/min/1.73 m^2^ categories were 0.882 (95% CI 0.845–0.914), 0.785 (95% CI 0.737–0.833), and 0.936 (95% CI 0.905–0.960), respectively. The model showed stable performance in the testing set, with AUCs of 0.859 (95% CI 0.804–0.910), 0.679 (95% CI 0.589–0.760), and 0.901 (95% CI 0.848–0.946) (Table 2 and Fig. 4a–b). The confusion matrices for each category in both the training and testing sets are presented in Fig. 4c–d. The calibration curves for the kidneys in each group closely align with the diagonal, indicating a good model fit (Fig. 4e–f). Furthermore, the clinical decision curves for each group of kidneys demonstrate varying degrees of clinical net benefit (Fig. 4g–h).

**Table 2:** The predictive performance of the radiomics model for three groups of SRF

	Training set			Testing set		
	> 45 ml/min/1.73m^2^	30–45 ml/min/1.73m^2^	< 30 ml/min/1.73m^2^	> 45 ml/min/1.73m^2^	30–45 ml/min/1.73m^2^	< 30 ml/min/1.73m^2^
Sensitivity	0.752(0.674–0.827)	0.594(0.500–0.681)	0.804(0.736–0.867)	0.640(0.519–0.769)	0.498(0.365–0.633)	0.756(0.656–0.862)
Specificity	0.847(0.796–0.890)	0.803(0.750–0.853)	0.931(0.898–0.960)	0.817(0.743–0.887)	0.746(0.667–0.827)	0.894(0.832–0.948)
Accuracy	0.816(0.774–0.856)	0.736(0.690–0.782)	0.887(0.856–0.916)	0.759(0.698–0.821)	0.666(0.593–0.741)	0.845(0.790–0.895)
F1 score	0.728(0.664–0.788)	0.589(0.515–0.662)	0.832(0.782–0.878)	0.632(0.528–0.732)	0.486(0.362–0.594)	0.773(0.693–0.850)
AUC	0.882(0.845–0.914)	0.785(0.737–0.833)	0.936(0.905–0.960)	0.859(0.804–0.910)	0.679(0.589–0.760)	0.901(0.848–0.946)

### The model performance in subgroups

**Fig. 5:**
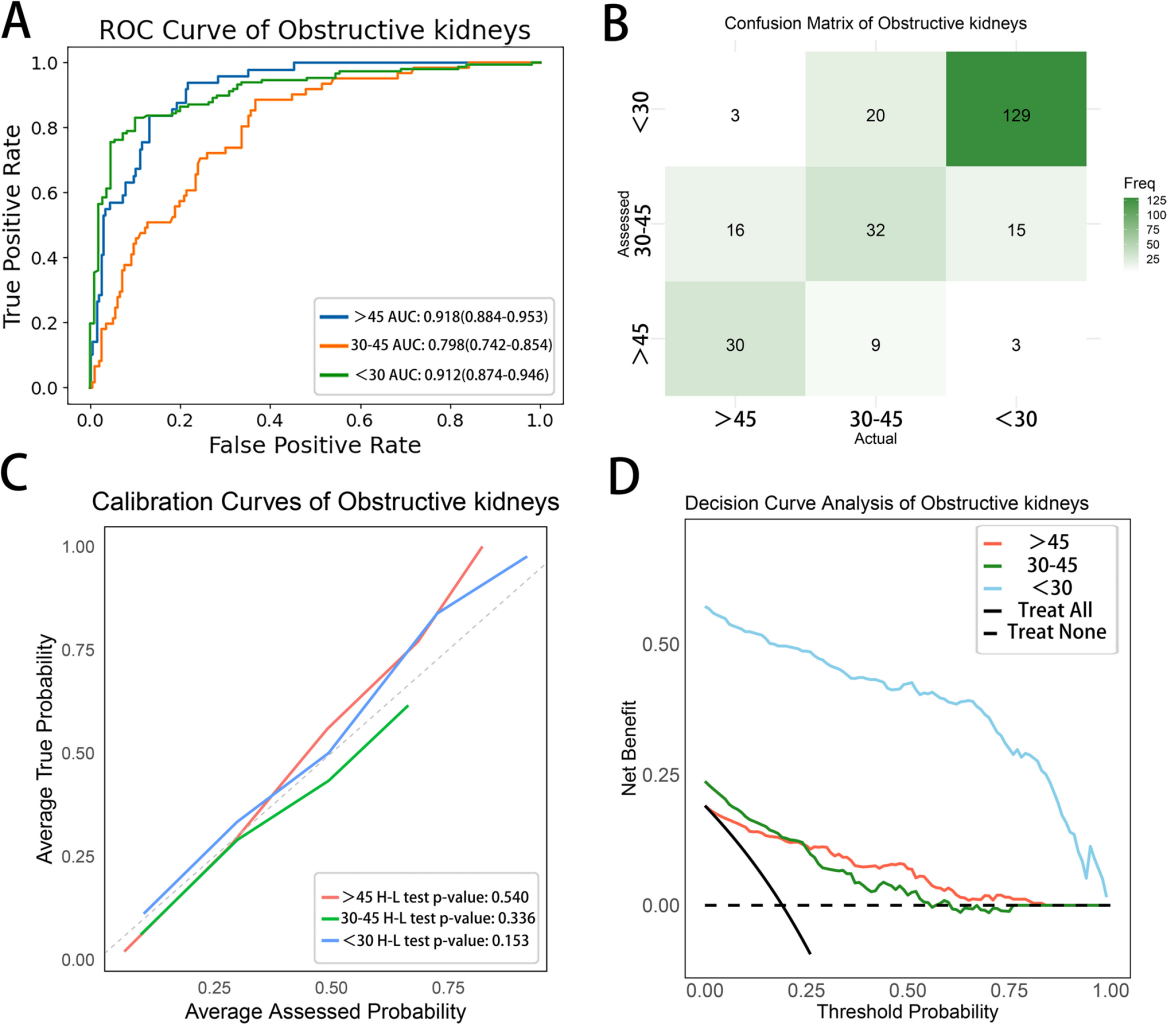
The performance of the radiomics model in obstructive kidneys. **a** ROC curve. **b** Confusion matrix. **c** Calibration curve. **d** Clinical decision curve analysis

Moreover, the model demonstrated good predictive ability and clinical utility across various subgroups, particularly in the obstructive kidneys group. In this subgroup, the AUC for the > 45 ml/min/1.73 m^2^, 30–45 ml/min/1.73 m^2^, and < 30 ml/min/1.73 m^2^ categories were 0.918 (95% CI 0.884–0.953), 0.798 (95% CI 0.742–0.854), and 0.912 (95% CI 0.874–0.946), respectively (Table 3 and Fig. 5).

**Table 3:** The predictive performance of the radiomics model for each subgroup

	Normal kidneys			Obstructive kidneys			Space-occupying kidneys		
	> 45 ml/min/1.73m^2^	30–45 ml/min/1.73m^2^	< 30 ml/min/1.73m^2^	> 45 ml/min/1.73m^2^	30–45 ml/min/1.73m^2^	< 30 ml/min/1.73m^2^	> 45 ml/min/1.73m^2^	30–45 ml/min/1.73m^2^	< 30 ml/min/1.73m^2^
Sensitivity	0.772(0.675–0.859)	0.600(0.469–0.733)	0.577(0.333–0.786)	0.614(0.478–0.744)	0.526(0.397–0.649)	0.878(0.823–0.929)	0.734(0.606–0.860)	0.578(0.448–0.700)	0.422(0.222–0.640)
Specificity	0.712(0.603–0.819)	0.757(0.670–0.843)	0.985(0.962–1.000)	0.943(0.912–0.972)	0.843(0.793–0.893)	0.791(0.718–0.866)	0.695(0.595–0.794)	0.667(0.561–0.775)	0.972(0.939–1.000)
Accuracy	0.745(0.667–0.811)	0.705(0.634–0.778)	0.934(0.895–0.967)	0.881(0.840–0.918)	0.767(0.720–0.821)	0.841(0.794–0.883)	0.708(0.632–0.789)	0.624(0.541–0.707)	0.873(0.812–0.925)
F1 score	0.767(0.688–0.836)	0.568(0.457–0.674)	0.679(0.471–0.839)	0.658(0.539–0.765)	0.517(0.414–0.619)	0.863(0.816–0.902)	0.629(0.515–0.736)	0.595(0.485–0.694)	0.538(0.320–0.727)
AUC	0.820(0.749–0.881)	0.727(0.644–0.805)	0.920(0.808–0.987)	0.918(0.884–0.953)	0.798(0.742–0.854)	0.912(0.874–0.946)	0.794(0.721–0.866)	0.651(0.551–0.739)	0.858(0.774–0.929)

Normal kidneys: Refers to kidneys that exhibit no significant abnormalities on imaging, such as kidney stones, hydronephrosis, renal cysts, or renal tumors

Obstructive kidneys: Refers to kidneys affected by upper urinary tract obstruction due to various lesions, including stones or tumors

Space-occupying kidneys: Refers to kidneys with cysts or tumors that do not result in urinary tract obstruction

## Discussion

Currently, there are limited methods for assessing SRF in clinical practice. The most commonly used RDI is constrained by factors such as low spatial resolution, complex procedures, and high equipment costs. In this study, we demonstrated that renal NCT radiomics can effectively reflect SRF information. We developed an SRF assessment model utilizing renal NCT, which offers advantages such as high spatial resolution, ease of operation, and cost-effectiveness. This model overcomes the limitations of RDI, providing a novel approach for the clinical evaluation of SRF.

This study classified renal function into three groups based on GFR and, for the first time, developed a radiomics-based model for SRF assessment using renal NCT. The model accurately distinguishes kidneys with GFR > 45 ml/min/1.73 m^2^ and GFR < 30 ml/min/1.73 m^2^, with AUC values of 0.859 (0.804–0.910) and 0.901 (0.848–0.946) in the testing set, respectively. However, the model performs less effectively in distinguishing kidneys with GFR between 30 and 45 ml/min/1.73 m^2^, with an AUC value of 0.679 (0.589–0.760) in the testing set. This may be because this group requires excluding both well-functioning and poorly functioning kidneys, which adds complexity to the model.

The model exhibited stable performance across different subgroups, demonstrating stronger applicability in obstructive kidneys. The AUC values for identifying kidneys with GFR > 45 ml/min/1.73 m^2^, GFR between 30 and 45 ml/min/1.73 m^2^, and GFR < 30 ml/min/1.73 m^2^ in this subgroup were 0.918 (95% CI 0.884–0.953), 0.798 (95% CI 0.742–0.854), and 0.912 (95% CI 0.874–0.946), respectively. This may be due to the more uniform parenchymal damage in obstructed kidneys, which is easier to detect [15]. This finding suggests that in obstructive kidney patients, NCT can be used not only to assess the severity of obstruction but also to assess renal function, allowing for timely intervention, reducing the need for RDI, and thereby saving both time and economic costs.

Several studies have previously investigated the value of CT radiomics in assessing chronic kidney disease patients. A multicenter prospective study [10] explored the relationship between the changes in renal parenchymal volume obtained from CT scans and radiomics features with kidney function deterioration in patients with autosomal dominant polycystic kidney disease. The study demonstrated good predictive performance, with AUC values of 0.752 and 0.849 for the renal parenchymal volume change rate and radiomics features, respectively, while the combined model achieved an AUC value of 0.902. Another recent study [16] involving 124 patients examined the identification of chronic kidney disease patients (GFR < 60 ml/min/1.73 m^2^) based on renal volume or NCT radiomics features. The results revealed that the model based on the renal volume had an AUC of 0.746, whereas the radiomics-based model achieved an AUC of 0.878. These studies indicate that CT radiomics features can be used to identify kidney function, but they primarily focus on overall renal function and do not evaluate SRF, limiting their applicability.

Furthermore, a recent study [17] investigated the feasibility of using magnetic resonance diffusion-weighted imaging radiomics to assess SRF. The study included 330 kidneys, categorized by GFR into normal (GFR > 40 ml/min), mildly impaired (20 ml/min ≤ GFR < 40 ml/min), moderately impaired (10 ml/min ≤ GFR < 20 ml/min), and severely impaired (GFR < 10 ml/min). The study constructed three radiomics models to differentiate normal from impaired, mild from moderate, and moderate from severe kidney dysfunction. The testing results showed that all three models exhibited reasonable discriminatory ability, with AUCs of 0.843, 0.717, and 0.897, respectively. This study confirmed the potential of diffusion-weighted imaging-based radiomics models in assessing SRF. While the findings of this study are similar to ours, it is worth noting that CT, as used in our research, is more accessible, easier to operate, time-efficient, and free from contraindications such as claustrophobia or metallic implants, in contrast to magnetic resonance imaging.

Numerous studies [18, 19] have confirmed that for patients with GFR < 30 ml/min/1.73 m^2^, the risk of mortality, cardiovascular events, kidney function deterioration, and hospitalization significantly increases. Some studies [20–22] further suggest that for patients undergoing radical nephrectomy, the long-term kidney function and survival outcomes are not significantly affected when the GFR of the contralateral kidney is > 45 ml/min/1.73 m^2^. The SRF evaluation model constructed in this study based on renal NCT radiomics features accurately differentiates kidneys with GFR > 45 ml/min/1.73 m^2^ and those with GFR < 30 ml/min/1.73 m^2^ across all kidneys assessed. The model's accuracy was further validated in subgroup analyses, demonstrating its potential utility in assessing the function of the contralateral kidney in patients planned for radical nephrectomy due to conditions such as obstructive nephropathy or tumors. This model offers valuable insights for guiding clinical treatment decisions, assessing patient prognosis, and predicting disease progression.

Despite its strengths, this study has several limitations. First, the classification of renal function into three categories based on GFR limits the ability to achieve a more precise quantification of SRF. Secondly, while the results from the test set suggest that the model demonstrates good generalizability, the study lacks external validation, as it is a single-center retrospective investigation. Future multicenter studies with larger sample sizes are needed to further validate the model's value.

## Conclusions

This study confirms that renal NCT radiomics features effectively reflect SRF information. The three-class random forest assessment model constructed using these features demonstrates strong assessment performance, particularly in accurately distinguishing between kidneys with GFR < 30 ml/min/1.73 m^2^ and those with GFR > 45 ml/min/1.73 m^2^. This model offers a novel approach for the clinical assessment of SRF, providing a simpler, more economical alternative to RDI, as renal NCT offers high spatial resolution, enabling the provision of morphological structural information while simultaneously reflecting SRF, thus holding significant promise for clinical applications.
